# The Application of Deep Learning Human Pose Estimation in Sport: A Systematic Review

**DOI:** 10.1186/s40798-025-00953-3

**Published:** 2025-12-10

**Authors:** Cavan Aulton, Lois Wakili, Ben William Strafford, Keith Davids, Chuang-Yuan Chiu

**Affiliations:** School of Sport and Physical Activity, College of Health, Wellbeing and Life Sciences, Sheffield Hallam University, Collegiate Hall, Collegiate Crescent, Sheffield, S10 2BP UK

**Keywords:** Human pose estimation, Deep learning, Movement skill analysis, Action recognition, Augmented coaching tools, Officiating sport

## Abstract

**Background:**

Human Pose Estimation (HPE) has gained increasing attention in sports research due to advancements in Deep Learning (DL) movement skills, which enable precise joint localization in 2D and 3D visual data. DL-based HPE facilitates non-invasive analysis of movement patterns in real-world settings, providing actionable insights for training, performance optimisation, and injury prevention. This systematic review examines the application of DL-based HPE in sports, focusing on the availability and accessibility of training datasets, reproducibility for practitioners, and the influence of human factors. The review also offers recommendations to guide future research and applications.

**Methods:**

A systematic search following PRISMA guidelines was conducted across four databases—Scopus, Web of Science, the Association for Computing Machinery, and SPORTDiscus, yielding 371 articles. Two independent reviewers applied inclusion and exclusion criteria to identify relevant studies, with a third reviewer resolving conflicts. Key aspects analysed included the scope of DL-based HPE applications, dataset characteristics, and algorithmic approaches. A supplementary search was conducted to include contemporary literature published since the initial search date. Data were synthesized descriptively, focusing on trends and limitations in the evidence base.

**Results:**

The identified applications of DL-based HPE in sports were categorized into four domains: movement skill analysis, action recognition, augmented coaching tools, and officiating support. Most studies relied on private datasets for algorithm training and validation, limiting reproducibility and generalizability. Bespoke multi-model algorithms were the most common approach, and single person pose estimation predominated. Despite its potential, the lack of open datasets and standardized practices poses challenges for broader adoption and practical implementation. These findings were echoed in the supplementary search which added no significant findings outside what previous studies had demonstrated.

**Conclusions:**

This review represents the first systematic evaluation of DL-based HPE from a sports science perspective, offering practical guidance for future research and applications. The findings highlight the need for open, standardized datasets and reproducible methodologies to advance the field. Future research should address these limitations while exploring innovative applications to maximize the impact of DL-based HPE in sports science.

## Key Points

- The application of Deep Learning (DL) Human Pose Estimation (HPE) in sport can be categorised into four application domains: movement skill analysis, action recognition, augmented coaching tools, and officiating support.
- A major disconnect exists between technological advancement and its practical application, largely due to barriers in accessibility and reproducibility. The widespread use of private datasets and bespoke algorithms makes most methods inaccessible to sports practitioners, highlighting the need for large, open-access, and context-rich sporting datasets.
- A crucial finding is the complete absence of longitudinal studies that empirically test the long-term effects of implementing DL HPE tools in athlete development programs. Without evidence that these technologies lead to tangible performance gains, practitioners may be reluctant to adopt them.

## Introduction

Human Pose Estimation (HPE) is a method of estimating the position of different body parts during movement from images and videos [1]. Two-dimensional (2D) HPE can estimate the position or spatial location of joints and bones from 2D images or videos [2], whilst three-dimensional (3D) HPE aims to estimate the position and orientation of joints and pose of the body from single or multiple 2D images or videos [3]. Deep learning (DL) is one approach to Machine Learning (ML) and allows HPE algorithms to learn from datasets, allowing them to recognise similar scenarios when presented with them in the future [4]. A dataset used for training DL HPE models is a collection of annotated images and videos containing a variety of general poses (i.e., walking, crouching, and jumping) or sport-specific poses allowing the model to accurately learn various scenarios. While standard 2D colour (RGB) images are the most common input, these models can also be trained using other data modalities such as 3D depth maps or pre-processed skeletal coordinates [3].

DL has emerged as the superior approach to HPE compared to the traditional approaches because of its ability to capture more detailed poses and provide more accurate tracking. Feature extraction (FE) based HPE methods rely on handcrafted features to detect the body in different postures; however sport is dynamic, and athletes often generate unique poses so many features are required to create accurate and robust FE HPE algorithms [5]. FE methods use spatial relationships between body parts and summarise predictions into a single value which restricts the accuracy and detail of their predictions [5]. Unlike FE, DL HPE does not require individual features and instead works in a unified manner, considering all body parts simultaneously. This allows DL approaches to find the most accurate solution to tracking, which has an associated increased computation cost [5]. Practitioners want tools that are fast and efficient and can be seamlessly integrated into their athlete development programs. DL HPE offers this as it does not require constant manual feature adjustments like FE approaches which is a time saving and financial benefit to sporting organisations. Therefore, DL HPE forms the focus of this systematic review.

HPE is a popular research area and has a variety of applications within sport, defined as a physical activity that is typically organised, competitive and can be conducted individually or as a team [6]. It should be noted that HPE in its current state is unable to provide fixed point tracking data in the way that 3D motion capture systems can. As the name states, HPE only estimates joint locations in visual data, but year on year joint detection and tracking accuracy are improving [7]. However, the application of HPE can deliver a variety of advantages for sports practitioners such as providing a deeper understanding of action in real time or reducing practitioner workload by forming the basis of an automatic notational analysis tool [8].

The systematic identification of gaps within existing literature can play a vital role in steering the future direction of research, enabling and guiding novel studies that support the continual evolution of DL HPE in sport. Supporting the advancement of these technologies through the identification of gaps can ultimately enhance the effectiveness and applicability of DL HPE in sport. Reviewing literature surrounding DL HPE in sport can also offer valuable guidance to applied practitioners by demonstrating how accessible and effective the technology is and promotes the diverse and immediate application of DL HPE in sport. A systematic review offers a robust and reproducible method for collating and analysing literature relating to DL HPE in sport, providing a comprehensive overview of the current trends and knowledge gaps to researchers in a methodology favoured in academic research [9]. Outlining current and potential DL HPE applications for practitioners in sport and, by appraising published findings and datasets, whilst demonstrating the accessibility and reproducibility of DL HPE systems, can support future applications of these technologies.

Despite the growing body of literature surrounding DL HPE there is a significant disconnect between technological advancements and the practical guidance and implications for the end-users in the sporting world. For example, a previous review focused on HPE more broadly (i.e., not in sport) and on the algorithm architecture of available models and its performance in language and metrics directed at developers (i.e., percentage of direct key points (PCK)). Therefore, practitioners such as coaches, biomechanists, and sports scientists are left without a consolidated resource that evaluates these tools from an applied perspective. This leaves key questions concerning the accessibility of this technology, the reproducibility for research findings in applied practice, and the impact of critical human and contextual factors that remain unexplored in the current literature.

### Previous Reviews

Currently, there is a lack of comprehensive systematic reviews on DL HPE within the existing literature, particularly in the realm of sports. The limited systematic reviews that do exist focus on HPE and concentrate on a technological perspective, such as the different algorithmic methodologies, model architecture and limitations associated with these approaches. This gap in the published literature needs to be addressed to support the future application of DL HPE in sport. A previous review outlined the different approaches of conducting 3D HPE (i.e., discriminative, part based, and hybrid approaches), but limited discussion centred around DL due to its infancy at that time [10]. The authors outlined how current approaches to 3D HPE performed poorly in the real-world and recommended further technological developments in this area. Another review summarised the current state of DL 3D HPE and highlighted the need for development of better multi-person 3D HPE systems after identifying that 3D HPE performance suffers in human–human interactions due to occlusions which are commonplace in sport [10]. The only remaining review showed the different methodologies behind HPE with a focus on performance metrics and model structures and outlined the high computational cost of current approaches to HPE [11]. Given the high computational cost, the authors recommend the development of lightweight models that are more appropriate for use in an applied performance setting. The existing reviews lack a specific focus on sport, whilst directing their recommendations towards technology developers rather than addressing the practical needs of applied practitioners. This limitation of existing research creates a gap concerning the provision of actionable recommendations for practitioners aiming to enhance athlete development programs through the application of DL HPE systems.

At the time of writing only one review focused its scope on applications of HPE in sport [2], sought to examine the applications of HPE, along with the accessibility of training datasets, and assessed the reproducibility of these approaches in sport and physical activity. Similarly to previous reviews, this review outlined how current approaches have a high computational cost and recommended the development of lighter models that are more appropriate in an applied setting [2]. They also stated that use of private datasets reduced reproducibility of the findings of current approaches. Overall, previous reviews have focused on illustrating the technical specification of the models and providing recommendations targeted at developers, rather than outlining the current or potential applications of HPE to practitioners, and nor did they provide practical recommendations that can support applied practitioners in future applications of HPE in sport. Finally, the findings of previous reviews lack contextualisation within a theoretical framework to guide the future design and implementation of DL HPE.

Therefore, this systematic review focuses on application strategies over technological methodologies for the benefit of sport practitioners. This will help demonstrate to sport practitioners the current and potential applications of HPE, whilst maintaining a focus on the reproducibility and accessibility of current methodologies with the aim of fostering future application of DL HPE in sport. In doing so, this review offers a more distinct and targeted contribution than the previous systematic reviews [2, 13] by differentiating our work in three key ways. Firstly, by adopting a dedicated sports science perspective that exclusively includes sporting contexts and utilises sports-centric databases; secondly, by creating a novel practitioner-focused analysis that categorises applications into four practical domains based on their application characteristics (movement skill analysis, action recognition, augmented coaching, and officiating support). Additionally, by focusing on DL HPE we are helping guide practitioners to apply tools that are accurate and reliable which may not be the case in previous reviews that assess all HPE techniques even though many are completely inaccessible to applied practitioners due to the time-consuming nature of development [13]. Thirdly, by systematically analysing the impact of human and contextual factors, we place athletes and coaches at the centre of the technological application to maximise its real-world implementation.

### Human Factors

Human factors in the application of HPE in sport include the environmental settings present in the data the HPE system has been trained on or applied in (i.e., controlled non-representative environments or contextual sporting environments). Systems trained on data containing environments like laboratories, which lack features such as crowds, weather, and other players, may perform poorly in actual performance settings [12]. Additionally, the number of participants can affect HPE robustness, as systems may struggle with multiple individuals in the frame [13]. The skill level of participants could also impact HPE accuracy, influenced by movement speed and range [14]. For example, elite sprinters' high hand velocities can cause video occlusions, reducing accuracy [15]. All these factors are important to consider in order to ensure the effective application of HPE in sport.

Systematic reviews to date have not considered these human or contextual aspects of the application of HPE in sport, including participant and dataset characteristics (i.e., crowds, weather, and background complexity). Also, previous reviews tend to adopt a computer science perspective, omitting sports science databases from their literature search, causing them to miss valuable insights into the methodologies and applications of HPE in sport [2, 10–13]. Conducting future reviews from a sports science perspective can shift the focus to the applications of DL HPE in sport which will be more beneficial to applied sports practitioners. Finally, since a recent review was published a literature search using the exact terms from that review on Scopus using the search terms (“Pose Estimation”) AND (“Sports”) yielded around an additional 200 papers [2].

### Reproducibility and Accessibility

If algorithms and their associated training data remain private, then these approaches are not accessible to applied practitioners which hinders understanding in the future applications of these technologies in sport. Furthermore, the private nature of algorithms and datasets also means that developers and researchers may be unable to reproduce the results in published approaches to DL HPE in sport. Therefore, future systematic reviews should appraise the reproducibility and accessibility of the currently available DL HPE systems in sport to support practitioners in the application of these technologies in athlete development programs and guide future research.

### Potential Applications

The increase in publications necessitates a refreshed systematic review to thoroughly examine the literature on DL HPE in sports, whilst crucially addressing the shortcomings of prior systematic reviews. Offering applied practitioners’ insights into the latest advancements and potential applications of these technologies in sports, and providing researchers with current trends in this field, empowers them with up-to-date information that can guide future research into DL HPE In sport. Therefore, this systematic review follows PRISMA guidelines and will provide an examination of the literature relating to the application of DL HPE in sport which can demonstrate the applicability of these technologies to applied practitioners across sport.

### Differentiation from Previous Reviews

This review addresses the shortcomings of previous surveys, and Table 1 outlines the key points that differentiate it from that literature.

**Table 1:** Key points that will differentiate this review from previous literature

Difference	Explanation
1	This review will explores the potential applications of currently available DL HPE algorithms in sports, shedding light on the benefits these applications can offer to both practitioners and individual athletes. By focusing on DL HPE movement skills from a sports science perspective, it addresses a significant gap in the current literature. It not only evaluates the current utilisation of DL in sport, but also extends the discussion to future research directions, providing valuable insights for the application of HPE in sports
2	This comprehensive review aims to collate and understand the multifaceted human factors (see Human Factors) associated with the application of DL HPE in sport. By investigating these factors and the application of HPE, our objective is to identify prevailing trends, limitation, and potential advancements in the application of DL HPE in sports
3	This review examines whether the current approaches to DL HPE in sport are reproducible and accessible (i.e., public availability of algorithms and datasets and hardware requirements) to practitioners; if they are not, this could affect the future applications of HPE in sport

### Research Questions

This review is structured around the key research questions detailed in Table 2, each formulated to address a specific gap in the existing literature.

**Table 2:** Research questions that will be answered in this systematic review

	Research question	Purpose
1	What are the current and potential applications of deep learning HPE in sport?	Summarising current and potential applications of HPE in sport and can identifying gaps in the current applications of DL HPE. This can help guide researchers and practitioners in the future application of HPE in sport
2	Are currently available HPE systems accessible to practitioners?	Assessing the accessibility of HPE systems enables practitioners to make informed decisions regarding the future applications of DL HPE in sport, whilst highlighting to developers the specific methods of HPE that require increased accessibility
3	What are the human factors in the application of deep learning HPE in sport?	Outlining the human factors (see Human Factors) in the current applications of HPE in sport provides contextualised guidance to practitioners and researchers to promote a richer application of HPE in sport

## Methods

This section outlines the methodology that was followed throughout the systematic review including how data sources were selected and how the data were analysed.

### Inclusion and Exclusion Criteria

The inclusion and exclusion criteria set out in Table 3 allow this review to achieve its aims of appraising the literature surrounding the application of DL HPE in sport.

**Table 3:** Inclusion and exclusion criteria used in this systematic review

Variable	Criteria
Date	Only papers from 2014 to 2023 (present) are included in this review because 2014 was the advent of the use of Deep Learning in HPE
Publication type	Only journals and conference papers published in the academic databases searched in Table 4 have been included
Modelling type	For this review, only full body pose estimation is included. Following the precedent set in previous reviews, analyses focusing on human-object interaction, singular limbs, hands, or eyes are excluded [2]
Modelling method	Only Deep Learning approaches to HPE are included due to their popularity and robustness in comparison to traditional or feature-based methods of HPE
Language	Only English language papers are included in this systematic review
Setting	Only papers which applied DL HPE in sporting contexts are included, which means the removal of any physical activity, yoga, or dance papers. Papers tested on databases and/or applied to real-life participants are included

### Search Strategy

To search for papers on the application of HPE in sport during this systematic review the search terms were as follows: ((“Deep Learning") OR ("Markerless") OR ("Machine Learning") OR ("Neural Networks") AND (“Computer Vision”) AND (("Pose estimation") AND ("Sports")), and the search was limited to title, abstract and keywords of papers. Deep learning, pose estimation and sports were the primary search terms but more specific computer science language such as markerless and neural networks were included in the search terms to avoid papers being excluded.

The following databases were searched for papers from January 2014 to December 2023: Scopus, Association for Computing Machinery (ARM) Digital Library and SPORTDiscus and Web of Science (WoS). These databases were selected in line with the scope of this systematic review to include papers both from a computer science and sports science perspective (see Table 4). References from previous systematic reviews on HPE estimation were screened to find additional papers.

**Table 4:** Characteristics of the databases used for literature searching in this systematic review

Database	Description	Relevant topics
Scopus	Multi-disciplinary research database of peer-reviewed journals	Information technology, life sciences, medicine, healthcare, and engineering
SPORTDiscus	Comprehensive full-text research database of peer-reviewed papers on Sports and Exercise Science	Sports and Movement sciences, Sports medicine and exercise kinesiology
Association for Computing Machinery Digital Library	Research database on publications about computing and information technology	Computer science, Machine learning, Computer vision and artificial intelligence
Web of Science	Multi-disciplinary research database focused on scientific information	Computer science, Sports science, and information technology

To bring this review into line with contemporary literature (i.e., papers published since January 2024) a supplementary search using the original search terms and original databases was conducted. The aim of this search was to identify if any impactful research since this review was originally conducted and to appraise across time-points whilst not conducting an additional systematic review. However, to ensure the robustness and validity of the original PRISMA search, the full review process was not conducted in its entirety again.

### Study Selection and Reliability

Following the database search the articles were imported into Covidence (a systematic review management software) for the sampling process. After automatic removal of duplicates the two-stage screening process occurred. To ensure inter-rater reliability and minimise selection bias, two reviewers independently screened the titles and abstracts of the remaining papers against the predetermined inclusion and exclusion criteria (see Table 3). Disagreements on study selection were resolved through discussion and if consensus could not be reached a third independent reviewer provided the final decision. This process was repeated for the full-text review of the remaining studies to finalise the articles for inclusion.

### Data Extraction

For all studies that met the final inclusion criteria, the principal investigator extracted data into a predefined spreadsheet. A second reviewer then independently checked the extracted data for accuracy and completeness. In this systematic review, 11 metrics were extracted from the included studies: Sport, Participants, 2D or 3D, Multi-person, Algorithm(s), Training Dataset(s), Validation Dataset(s), Dataset Characteristics, Validation Metric(s), and Practical Application. Details such as technical training parameters (e.g., learning rates, batch sizes) and other computer science-based metrics were omitted from the primary analysis in this review because of our aim to provide recommendations to sport science practitioners and not developers which has been the focus of a previous systematic review [2].

## Results

### Study Identification

**Fig. 1:**
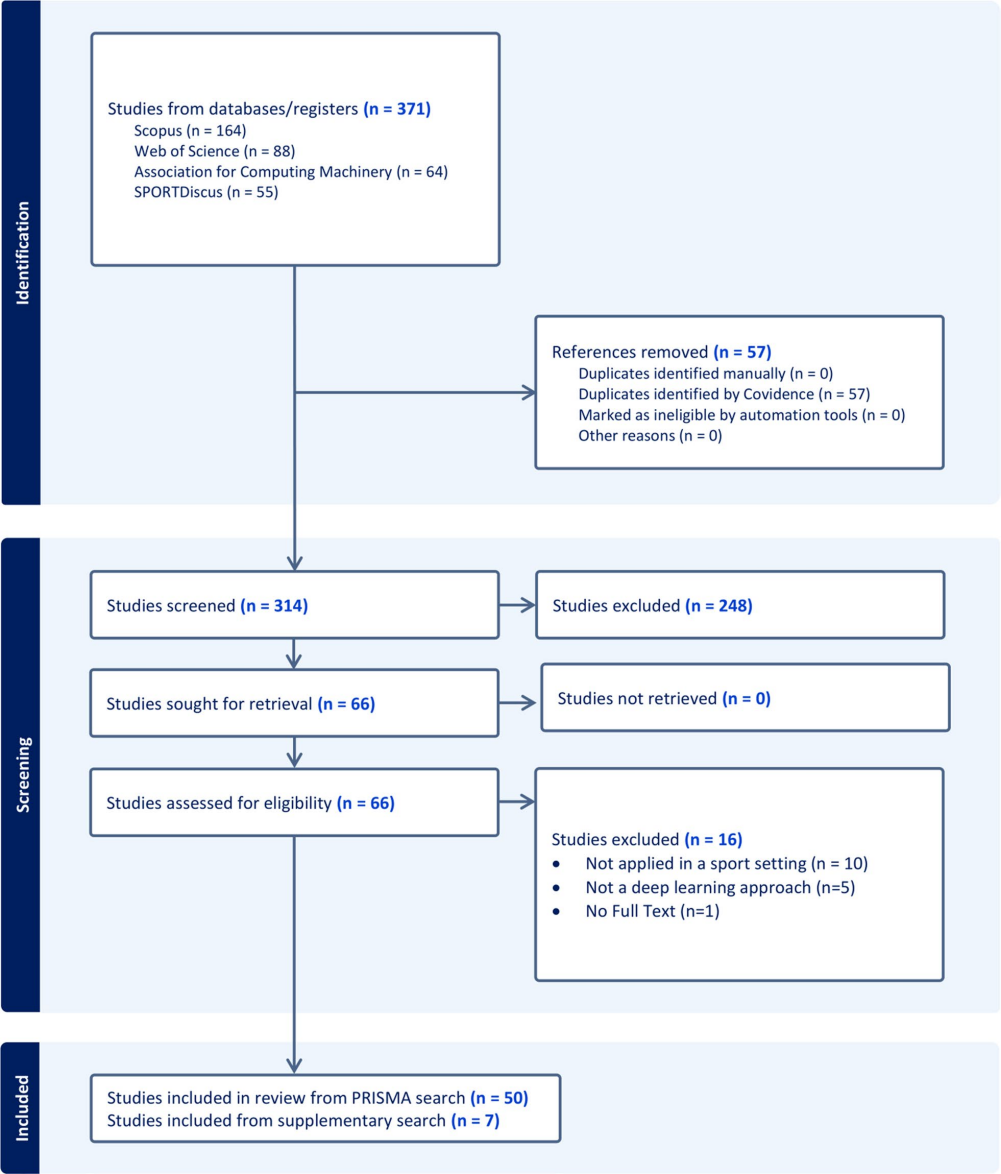
PRISMA flowchart outlining the paper selection process undertaken within this systematic review

Upon completion of the database search a total of 371 papers were found (Scopus = 164, Web of Sciences = 88, Association for Computing Machinery = 64, SPORTDiscus = 55), and no papers from additional sources were included. Covidence eliminated 57 duplicates from the subsequent analysis, and the remaining 314 papers underwent independent screening by two reviewers, who assessed their relevance based on the titles and abstracts using the inclusion and exclusion criteria outlined in Inclusion and Exclusion Criteria. Following this screening procedure, 66 papers were included in the full text review carried out again by the two independent reviewers. After the review process, 50 papers were included in this systematic review (see Fig. 1). The supplementary search identified only 7 additional relevant papers, all of which were found in the Scopus database.

### Descriptive Statistics

**Fig. 2:**
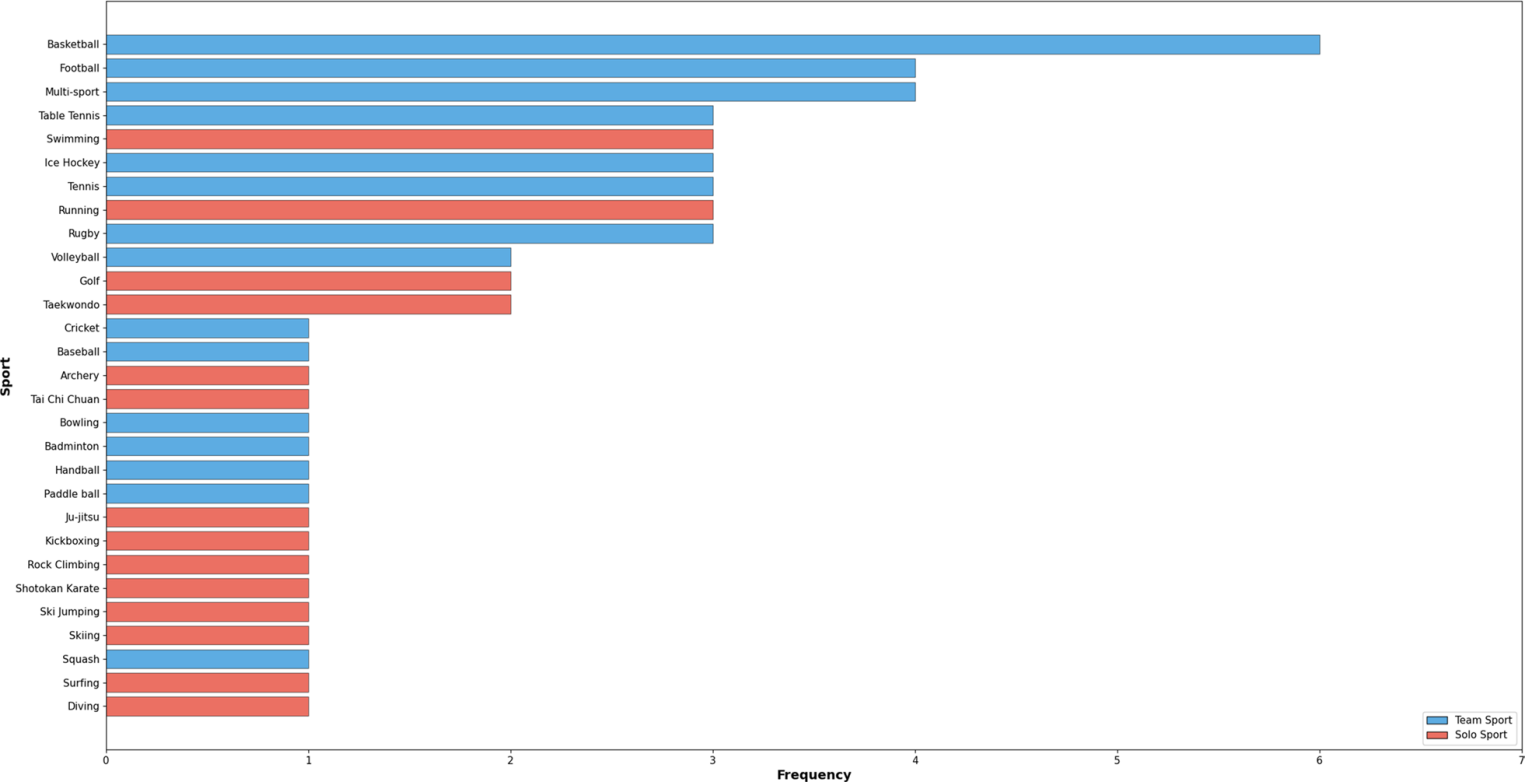
Frequency bar chart illustrating the various types of sports and the corresponding number of occurrences in the research papers included in this systematic review

After the 50 papers were exported into Excel for further analysis it was discovered that there were 20 2D studies and 27 3D studies with the remaining 3 being algorithm comparison studies that contained both 2D and 3D algorithms. Most of the studies classed as 3D HPE used an additional model such as monocular depth estimation, DL regression, or inverse kinematics to convert their 2D approach into 3D HPE. Only 15 studies focused on the more complex multi-person detection algorithms, with the remaining 35 papers focused on the use of single person detection algorithms. From a human factors perspective, out of the 50 papers included, only 12 validated and evaluated their DL HPE algorithms on live human participants with the remaining 38 papers choosing to validate their papers using datasets only. Figure 2 outlines the wide variety of sporting contexts DL HPE was applied in, with basketball being the most popular application of DL HPE. OpenPose was the most popular HPE algorithm used in 14 of the studies included within this review. Additionally, the most popular option for training and validation was with a private dataset and occurred in 21 studies in this review. Overall, the studies extracted in this systematic review can be categorized into four types of application of DL HPE in sport: (1) Movement skill analysis sits within the broader field of ‘performance analysis’ and is used to understand how sports skills are performed, providing a basis for improving performance, and these applications are shown in Table 5; (2) tactical or notational analysis studies the number of actions performed during training or competition and evaluates how they play a crucial role in athlete development programs, forming the basis to improve athletes during and post-performance [16]. Notational analysis includes an analyst counting how many times an action occurs during the game, and the studies contributing to the automation of this process are listed in Table 6; (3) Table 7 outlines the augmented coaching tools, which combine performance-context and computer-generated content to provide individualised performance feedback in the home or in training environments without the need for a coach [17]; (4) DL HPE systems that can support the officiating of sport, typically carried out by referees, umpires, and judges and tasked with maintaining fairness, reducing injury and applying the rules that govern a game or sport in an unbiased manner, are outlined in Table 8 [18]. The 7 additional papers found in the supplementary search reflected similar trends to the main review. Those studies favoured 3D HPE (4 out of 7 studies) and a heavy reliance on bespoke algorithms (6 of 7). Finally, single-camera approaches (6 of 7) were the most popular, with testing on datasets again being the preferred methodology (6 of 7). These supplementary papers are appraised separately in Table 9.

**Table 5:** Studies that used DL HPE for pose landmarking and movement skill analysis in sport

Study	Sport	Participants	2D or 3D	Multi-person	Algorithm(s)	Training dataset(s)	Validation dataset(s)	Dataset characteristics	Validation metric(s)	Practical application
Bachmann et al. [19]	Skiing	x'	3D	x'	OpenPose + Gaussian smoothing	Private dataset	Private dataset	Variety of weather patterns, participants and training tasks but contains a small amount of data	MPJPE + MAE + PCK	Allows the quantification of biomechanics variables on unknown orientation cameras
Baclig et al. [20]	Squash	x'	2D	x'	General-purpose multi-person pose estimation neural network	MPII human multi-person + COCO 2016 keypoint challenge dataset	Private dataset	Annotated competition videos in this dataset meaning it is highly representative of the performance environment	Correlation and max absolute difference to ground truth	Automatic performance analysis tool for squash
Duan et al. [21]	Basketball	x'	3D	x'	OpenPose + HRNET Feature Map	COCO2017	COCO 2017	This public dataset consist of 330 k images in a variety of environments and participants and with 200 k annotated images but is not sport specific	AP + mAP + AR	Improve player tracking methods in basketball non-invasively
Giulietti et al. [22]	Swimming	Male and Female (elite level)	2D	x'	SwimmerNET (multiple FCN-inspired architectures)	Private Dataset	x'	A representative training dataset of elite swimmers was collated but only 2021 frames are included in the dataset	MPE + % not recognised	Provides athletes and coaches a better understanding of performance during training
Groos et al. [23]	Multi-sport	x'	2D	x'	Efficient pose	MPII	MPII validation dataset	This dataset contains a large amount of sport specific training data in a variety of environments	PCK	Could be adapted into a technique analysis system but currently just an assessment of how accurate the model is from a computer science perspective
Hu [24]	Football	x'	3D	x'	DetectNet + 2D PoseNet + 3DPoseNet	MPII + MSCOCO + LSP	LSP + MSCOCO + MPII	These datasets contain a large amount of sport specific training data in a variety of environments	AP % + MPJPE	Inexpensive method to obtain player positional data for players without the need for addition sensors
Javadiha et al. [25]	Paddleball	x'	3D	x'	Comparative study	Comparative study	Private dataset	This private dataset contained a variety of environments along with different skill levels, but was a small dataset taken from one camera angle	DR + AP	Inexpensive method to obtain player positional data in amateur paddleball clubs
Jiang et al. [26]	Golf	x'	2D	x'	GolfPose	Private dataset	Private dataset	A large dataset of 120,000 annotated images that includes a breadth of environment, weather conditions and participants characteristics	MPE	Non-invasive method of movement skill analysis in golf
Li et al. [27]	Baseball	10 males	2D	x'	OpenPose	x'	x'	No information on training dataset but the approach was validated in real-life participants during training on a competition standard baseball pitch with athletes hitting a stationary ball	Custom scoring system	Swing analysis to support skill acquisition of the baseball swing
Ludwig et al. [28]	Ski Jumping	x'	2D	x'	MobileNet + adapted Mask R-CNN + RANSAC model	Private dataset	Private dataset	Contained 10,070 images form 290 jumps from a variety of competitions, ski jumps, countries and weather environments and participant characteristics	PCK + PCA	Provides information on flight parameters in a non-invasive method
Murakami and Nakamura [29]	Tennis	x'	3D	x'	Mask R-CNN and T-CNN	Human 3.6 M dataset	Private dataset	Training was conducted using a diverse and representative dataset, whilst validation was conducted on a privately created dataset of publicly available competition videos	Variance of length between joints	Movement skill analysis and automatic performance analysis tool for tennis
Murthy et al. [30]	Diving	x'	2D	x'	T-CNN and DiveNet pose	DSV dataset + IAT dataset	DSV dataset + IAT dataset	The training and validation of this study was conducted on publicly available datasets contain a wider array of environments and participants characteristics	PCK	Enriches the understanding of the behaviour of CoM during diving
Needham et al. [31]	Running	12 athletes (7 males and 5 females)	3D	x'	OpenPose	COCO	x'	The dataset used training is the largest public training dataset created with a vast variety of movements, environment, participant characteristics and sport specific movements	Mean Differences + Coefficient of determination	Analysis of CoM behaviour during sprinting in a non-invasive manner
Neher et al. [32]	Ice Hockey	x'	2D	x'	HyperStackNet	MPII dataset	HARPE dataset	The 2 datasets used for training are publicly available and contain a variety of environments, sport specific and general movements, and participant characteristics	PCK	Potential to improve the understanding of locomotion of ice hockey players in a non-invasive manner
Ooke et al. [33]	Taekwondo	x'	3D	x'	HRNET	Private dataset	Private dataset	This small private dataset used for training consists of lab bases images without representative sporting scenarios, but the validation dataset consists of manual annotated competition videos	Reconstruction error (MM) + Precision (%)	Reconstructing movements in a digital 3D space can improve the coaches' understanding of performance
Šajina and Ivašić-Kos [34]	Handball	x'	3D	x'	Comparative study	Private dataset	Private dataset	This private dataset is small containing only 227 images and images are in a single environment with no opposition players which is not representative of the performance environment	PCK + MPJPE + AP	Could aid in the analysis of movement skill of a single athlete during performance
Siddiqui et al. [35]	Cricket	x'	3D	x'	Comparative Study	Private dataset	Private dataset	This small private dataset included 150 images of a variety of cricket movements with a variety of participants in representative equipment and environmental features	Accuracy (%) + K-Fold Validation	Could help improve coaching movement skills and enhance batsmen’s performance in cricket
Sun [36]	Golf	x'	2D	x'	OpenPose	x'	Private dataset	This dataset was created in a lab environment with 1 participants so although it contains 25,000 images it is not representative or diverse as seen in other databases	% of landmarks detected	Non-invasive method of movement skill analysis in golf
Zecha et al. [37]	Swimming	x'	2D	x'	DCNN	Private dataset	Private dataset	1200 images for training and 30 videos for validation, containing a diverse range of participants, but validations were all conducted in the same environment	PCK	Non-invasive stroke analysis tool for swimming
Zecha et al. [38]	Swimming	x'	2D	x'	CPM	Leeds sports dataset + private dataset	Private dataset	8532 annotated frames used for training and validation containing a wide variety of strokes, environments and participants which is representative of the training environment	PCK + PCKP	Novel method of performing motion analysis on swimmers without the need for markers
ZöLlner et al. [39]	Surfing	x'	3D	x'	Comparative study	Privately created dataset	Privately created dataset	Small dataset containing 1 participant from one angle in a manmade environment which is not representative of the performance environment	Plotting traces of model against ground truth	Aid in the instruction and analysis of surfing movement skill

Average precision (AP), Average Recall (AR), Action recognition Hourglass Network (ARHN), Artificial Neural Network (ANN), Body Feature Alignment Based on Pose (BFAP), Convolutional Pose Machine (CPM), Coefficient of Variation (CV), Deep Convolutional Neural Network (DCNN), Detection Rate (DR), Fully Convolutional Network (FCN), Fuzzy Neural Network (FNN), Graph Convolutional Neural Network (GCNN), High-Resolution Network (HRNET), Long Short-Term Memory (LSTM), Learnable Triangulation (LT), Mean Absolute Error (MAE), Mean Average Precision (MAP), Mean Per Joint Position Error (MPJPE), Mean Percentage Error (MPE), Mean Squared Error (MSE), Percentage of Correct Angles (PCA), Percentage of Correct Point (PCP), Percentage of Correct Key Points (PCK), Percentage of Correct Keypoints—Proximal (PCKP), Part-based Hierarchical Recurrent Neural Network (PHRNN), Piecewise Recurrent Neural Network (PRNN), Region-Based Convolutional Neural Network (R-CNN), Regional Multi-person Pose Estimation (RMPE), Root Mean Squared Error (RMSE), Recurrent Neural Network (RNN), Region Proposal Network (RPN), Standard Error of Estimate (SEE), Symmetric Mean Absolute Error (SMAPE), Single-Person Pose Estimation (SPPE), Spatio-Temporal Graph Convolutional Network (ST-GCN), Spatial Transformer Network (STN), Spatial–Temporal Relation Module (STRM), Temporal Convolutional Neural Network (T-CNN), Time Series Deep Neural Network (TSDNN), Temporal Segment Network (TSN), 3D Convolutional Neural Network (3DCNN)

**Table 6:** Studies that used DL HPE for action recognition in sport

Study	Sport	Participants	2D or 3D	Multi-person	Algorithm(s)	Training dataset(s)	Validation dataset(s)	Dataset(s) characteristics	Validation metric(s)	Practical application
Akan and Varli [40]	Football	x'	3D	x'	BFAP + ResNet50	SoccerNet Re-Identification Challenge 2022 dataset	SoccerNet Re-Identification Challenge 2022 dataset	A large representative dataset containing annotated validation and training data in a variety of environments, weather conditions and player characteristics	MAP	Can automatically track player actions in a football match based on pose
Fani et al. [41]	Ice Hockey	x'	2D	x'	ARHN	MPII dataset + Private dataset (HARPE)	Private dataset	The training dataset is a very large dataset that includes general and sport specific images, but the validation occurred on a small, single environment private dataset	% of correct poses classified	Enhances the understanding of frequency of actions in ice hockey
Janbi and Almuaythir [42]	Bowling	x'	2D	x'	MoveNET + BowlingDL	Private dataset	Private dataset	A small dataset containing 193 images in a variety of backgrounds, bowling alleys and participant characteristics	% of event classified	Classify and understand the usage of different bowling shots
Kulkarni and Shenoy [43]	Table Tennis	x'	2D	x'	TCN	COCO + Private dataset	COCO + Private dataset	The COCO dataset is a large set of annotated images of a variety of sport specific and general image data, whilst the private dataset offers a small amount of highly representative training data	Accuracy (%)	Automatic performance analysis tool for table tennis
Li et al. [44]	Tennis	x'	3D	x'	OpenPose + inverse transformer	UCF 101	UCF 101	Contains 13,320 short trimmed videos from 101 action categories in sport and general movements providing a variety of representative environments and training characteristics	Recognition rate (%)	Could improve the recognition effect of tennis actions and improve students’ learning and understanding of actions in the teaching process
Nandagopal et al. [45]	Multi-sport	x'	2D	x'	OpenPose + DCNN + RMSProp	MPII and COCO dataset	UCF Sport	Large amounts of publicly available training and validation data in a variety of environments with sport specific and general movement that include a variety of participants	F-score + Accuracy (%) + precision (%)	Could improve the understanding of human movement across multiple sports
Vats et al. [46]	Ice Hockey	x'	2D	x'	LSTM + CNN	MSCOCO + HARPET dataset	HARPET	MSCOCO contains 100 k annotated images including sport specific and general scenarios, whilst the HARPET dataset included a small amount of highly representative sport scenarios	Accuracy (%)	Provide more information on athlete movement during ice hockey games in a non-invasive manner
Xu et al. [47]	Volleyball	x'	3D	x'	Part-based hierarchical RNN (PHRNN)	Private dataset	Private dataset	A small private dataset of manually annotated images in a single noncompetitive environments with little variation in participants, clothing, or background	Detection rate (%) + Recognition rate (%)	Can automatically quantify the number of times an event happens in a volleyball match
Yang et al. [48]	Basketball	x'	2D	x'	CNN + LSTM	Private dataset	Private dataset	Over 10 k video segments from competitive basketball games including a large variety of events, backgrounds, and participant characteristics	Accuracy (%)	Automatic performance analysis tool for basketball
Zhang et al. [1]	Multi-sport	x'	3D	x'	RPN + DCNN	KTH + UCF sports datasets	MSCOCO dataset	Large and diverse publicly available datasets containing annotated images of a variety of situations with both sport specific and general movement present	Detection rate (%)	Automatic game scoring system reducing the number of officials required
Zuo and Su [49]	Basketball	x'	3D	x'	RMPE + DNN	Private dataset	Private dataset	The training dataset is sporting specific dataset containing 1200 videos of diverse and representative competitive environments	Recognition rate (%) + Accuracy (%)	Automatic action recognition that can be utilised as a performance analysis tool

Action recognition Hourglass Network (ARHN), Body Feature Alignment Based on Pose (BFAP), Convolutional Neural Network (CNN), Deep Convolutional Neural Network (DCNN), Deep Neural Network (DNN), Long Short-Term Memory (LSTM), Mean Average Precision (MAP), Part-based hierarchical RNN (PHRNN), Regional Multi-person Pose Estimation (RMPE), Region Proposal Network (RPN), Temporal Convolutional Network (TCN)

**Table 7:** Studies that applied DL HPE to create technology augmented coaching tools

Study	Sport	Participants	2D or 3D	Multi-person	Algorithm(s)	Training dataset(s)	Validation dataset(s)	Dataset(s) characteristics	Validation metric(s)	Practical application
Ait-Bennacer et al. [50]	Shotokan Karate	x'	3D	x'	OpenPose + FastPose	COCO Keypoint challenge + MPII Human Pose Dataset	Private dataset	Trained on a large multi-purpose dataset including an array of environments, scenarios, and participants characteristics, but validation was on a very small sport specific dataset with little variation	Recognition rate (%)	Can classify and rate karate movements and could help individuals learn skills without a coach
Akiyama and Umezu [51]	Baseball	4 Participants	3D	x'	OpenPose + CNN	x'	x'	No information on training set but was validated on a small number of real participants in non-representative conditions	Cosine similarity	Could support the of pitching a baseball without the need for a coach
Chao and Zhang [52]	Running	x'	3D	x'	DNN	Multiple datasets (not specified)	Private + FLIC + MPII dataset	A large amount of training and validation data from sport specific and general scenarios in a variety of environments with a breadth of participant characteristics	Accuracy (%)	Helps improve the understanding of movement during crouch starts
Jian et al. [53]	Badminton	15 participants (no more info)	3D	x'	MediaPipe (BlazePose)	Private (google owned) data	x'	Large dataset containing sport specific and general scenarios of a wider variety of people and environments	Qualitative user feedback	Classification of movements for learning and development in badminton
Nurahmadan and Pradnyana [54]	Taekwondo	x'	2D	x'	OpenPose	Private dataset	Private dataset	40 small videos used for training and validation in a single environment with limited participants characteristics	Accuracy (%)	Could be developed into a pose classifier to help aid in skill acquisition
Phang et al. [55]	Archery	9 experienced archers	3D	x'	RCNN + RPN	x'	x'	No information on training data but was tested on a small group of experienced archers in the same environment	Mean + SD of kinematic parameters	Support the learning of posture control in archery without the need for coaching input
Suda et al. [56]	Volleyball	2 Participants (3 years’ experience)	3D	x'	Kinect tracking	Privately created dataset	x'	A small non-representative with limited participants diversity used for training and only 2 real-life participants used for validation	RMSE	Return balls with additional information to aid in skill acquisition
Takeichi et al. [57]	Running	5 males	2D	x'	CPM	Privately created dataset	x'	No information on training data but was tested on 5 runners and compared to a wider set of running data of 642 participants	Correlation Coefficient (to ground truth data)	The application could be an easy and useful tool for running form analysis
Wang et al. [58]	Multi-sport	x'	3D	x'	ResNet-50 + STRM	Penn Action Dataset + Sub-JHMDB	Sub-JHMDB dataset + VOT2018-LT database + Freestyle Skiing Aerials dataset	Multiple large sport specific and general scenario datasets used to contain a wide variety of participant characteristics	PCK + F-score	Helps individual athletes learn new movement skills without the need for a coach
Wei et al. [59]	Tai Chi Chuan	x'	3D	x'	Yolov4 + TSDNN + PRNN + adapted XGBoost	Private dataset	Private dataset	Small non-representative training and validation set containing only 1 environment and 1 participant	MSE + MAE + SMAPE	Can teach Tai Chi Chuan in a home environment
Wessa et al. [60]	Kickboxing	x'	2D	x'	ANN	Privately created dataset	x'	Very small non-representative training and validation set containing limited environments and participants	Accuracy (%)	Could support the learning of kick boxing movement skills
Wu et al. [61]	Table-tennis	10 subjects (6 amateurs, 4 coaches)	2D	x'	CNN + LSTM	MPI3D, Human3.6 M, and privately created dataset	x'	Trained on large and diverse datasets containing general and sport specific scenarios and was validated on a small but diverse group of real participants	PCP + RMSE + Max error	Could benefit players in predicting ball path based on server pose
Shi and Hu [62]	Basketball	x'	3D	x'	PAFS + FNN	MPII COCO key point dataset	NtURGB + D120 dataset	Trained on large and diverse datasets containing general and sport specific scenarios	Detection rate (%)	Can train athletes to predict flight path based on take-off mechanics to improve interceptions and blocks

Artificial Neural Network (ANN), Convolutional Neural Network (CNN), Convolutional Pose Machine (CPM), Deep Neural Network (DNN), Fuzzy Neural Network (FNN), Long Short-Term Memory (LSTM), Mean Absolute Error (MAE), Mean Squared Error (MSE), Part Affinity Fields (PAFS), Percentage of Correct Point (PCP), Percentage of Correct Key Points (PCK), Piecewise Recurrent Neural Network (PRNN), Region-based Convolutional Neural Network (RCNN), Region Proposal Network (RPN), Root Mean Squared Error (RMSE), Standard Deviation (SD), Symmetric Mean Absolute Error (SMAPE), Spatial–Temporal Relation Module (STRM), Time Series Deep Neural Network (TSDNN)

**Table 8:** Studies that utilised DL HPE to support the officiating of sport

Study	Sport	Participants	2D / 3D	Multi-person	Algorithm(s)	Training dataset(s)	Validation dataset(s)	Dataset(s) characteristics	Validation metric(s)	Practical application
Blythman et al. [63]	Rugby	2 Participants	3D	x'	Learnable Triangulation (LT) model	Human3.6 M + COCO & MPII datasets	x'	A large amount of training and validation data from sport specific and general scenarios in a variety of environments but was validated in a lab environment	MAE + MPJPE	Potential to be used on video footage to measure the body kinematics leading to injuries in sporting collisions
Hudovernik and Skocaj [64]	Jiu-jitsu	x'	3D	x'	Private algorithm	Private dataset	Private dataset	2004 images for training and 492 for validation, a wide array of scenarios captured from multiple viewpoints but in a single environment with little participant variation	AP + AR	Reduced the number of officials needed for a competitive match that can also support human referees
Nishio et al. [65]	Rugby	x'	3D	x'	Comparative study	Private dataset	Private dataset	Large highly representative dataset containing 360 competitive matches with much variation	AUROC-score	A model which can identify high-risk events completely automatically using only videos as input to support officiating
Nonaka et al. [66]	Rugby	x'	3D	x'	CenterTrack and HRNet	Private dataset	Private dataset	750 images for training and 33 video clips for validation of competitive matches with a diverse range of scenarios	DR (%)	Automatically detect and classify high risk tackles in rugby to reduce injury risk and inform decision making

Area Under the Receiver Operating Characteristic Curve (AUROC), Average Precision (AP), Average Recall (AR), Detection Rate (DR), High-Resolution Network (HRNet), Learnable Triangulation (LT), Mean Absolute Error (MAE), Mean Per Joint Position Error (MPJPE)

**Table 9:** Studies found in the supplementary search with their practical application outlined

Study	Sport	Participants	2D/3D	Multi-person	Algorithm(s)	Training dataset(s)	Validation dataset(s)	Dataset(s) characteristics	Validation metric(s)	Practical application
Cardenas et al. [67]	Rock Climbing	x'	2D	x'	Beta Caller, YoloV8 and VITpose	Private Dataset	Private Dataset	4,100 images collected as image sequences from over 250 videos	Acc and RMSE	**Augmented coaching tool:** Help visually impaired climbers and teach novice climbers optimum route selection
Fang et al. [68]	Football	x'	2D	x'	Bespoke CNN and RNN model	Private Dataset (from Soccer-v3)	Private Dataset	500 full broadcast matches	Acc (%), Prec (%), and Rec (%)	**Action recognition:** Practical applications are limited as it cannot provide officiating advice and only predicts if there is a foul or not and does not consider the severity
Ren [69]	Table Tennis	x'	3D	x'	GCNN and Yolov8	NTU-RGB + D and COCO	NTU-RGB + D and COCO	Large action recognition datasets with millions of images	Acc, Rec, MCC and mAP	**Action Recognition:** This offers practical insights into athletes’ technique during specific movements but requires steps to be used as a full coaching tool
Shoaib and Husnain [70]	Football	x'	3D	x'	Openpose, TSN and 3DCNN	SoccerNet and Sports 1-m	SoccerNet and Sports 1-m	Annotated football match footage with detailed temporal annotations	MAP, Acc, and Rec	**Action Recognition:** This can form the basis of an automatic performance analysis software
Ye [71]	Basketball	x'	3D	x'	RMPE, STN and SPPE	UCF Basketball Dataset	UCF Basketball Dataset	Large broadcast based of basketball games	Accuracy	**Action Recognition:** This can form the basis of an automatic performance analysis software
Yu [72]	Tennis	x'	2D	x'	R-Cnn, PoseNet50, and ST-GCN	MSCOCO2017 Dataset and 4 others	MSCOCO2017 Dataset and 4 others	Large action recognition datasets with millions of images	Accuracy and MAP	**Action Recognition:** This can form the basis of an automatic performance analysis software or augmented coaching tool
Zheng et al. [73]	Basketball	1 Participant	2D	x'	RTMPose	Private Dataset	x'	2300 manually annotated 3 × 3 basketball game segments from 98 official games recorded during the China Dragon 3 × 3 Super League in 2024	CV, SEE	**Move Skill Analysis:** This tool can be used to quantify physical movements on the court but is far from being a usable tool

Accuracy (Acc), Convolutional Neural Network (CNN), Coefficient of Variation (CV), Graph Convolutional Neural Network (GCNN), Matthews Correlation Coefficient (MCC), Mean Average Precision (mAP), Precision (Prec), Recall (Rec), Regional Multi-person Pose Estimation (RMPE), Root Mean Squared Error (RMSE), Recurrent Neural Network (RNN), Region-based Convolutional Neural Network (R-Cnn), Single-Person Pose Estimation (SPPE), Standard Error of Estimate (SEE), Spatio-Temporal Graph Convolutional Network (ST-GCN), Spatial Transformer Network (STN), Temporal Segment Network (TSN), 3D Convolutional Neural Network (3DCNN)

## Discussion

This systematic review aimed to analyse studies that applied DL HPE in sport, collating the current and potential applications of the technology, assessing the reproducibility of current methods of DL HPE, and outlining the human and contextual factors relating to its application in sport. While quantitative meta-analysis reporting combined effect sizes is a powerful tool for synthesis, the nature of the current literature makes such an analysis infeasible. A central finding of this review is the notable absence of intervention studies that would report effect sizes. Contrastingly the included studies overwhelmingly reported technical validations metrics (PCK, MPJPE, and mAP) which are standard for computer science research. These metrics are standard for assessing algorithmic performance, but they are inaccessible and lack direct practical interpretation for sports science practitioners. For example, practitioners are less concerned with a specific mAP score and more with whether a system is trustworthy and 'accurate enough' for informing their decision-making process.

We synthesise our findings in a theoretical framework grounded in ecological dynamics (ED) and the constraints-led approach (CLA) which aims to improve the application of machine learning and computer vision in sport [74]. The ED framework views skilled behaviour as emerging from the relationship between a performer and their environment. The CLA operationalises this by considering how individual, environmental, and task constraints shape movement solutions. This allows us to appraise not just technical metrics, but also whether these applications are ecologically valid and sufficient for practical use. This theoretically informed approach bridges the gap between the technical literature and the end-user, providing a synthesis of knowledge to support the effective future implementation of DL HPE in sport.

The results of this systematic review show that 3D HPE is preferred over 2D HPE. This preference may be due to the desire for more information on locomotion in all three planes (x, y, z), giving practitioners a deeper understanding of athlete performance, though this has yet to be empirically proven [74]. Most 3D algorithms utilised an additional layer to convert 2D approaches into 3D HPE, affecting accuracy due to reconstruction error. However, this approach is more suited to real-world application (i.e., only one camera) and can be applied retrospectively. Multi-person HPE was the least common (15 out of 50 studies), which supports the findings of previous systematic reviews [3]. However, most sports involve dynamic interactions between multiple athletes so focusing on a single athlete's performance can remove contextual factors relating to performance [75]. This complexity and computing power requirement may be addressed by the creation of cloud-based HPE systems.

Participant validation in performance contexts can be defined as researchers applying their DL HPE systems with participants in their training environments as they perform, whereas database only validation can be defined as validating DL HPE systems retrospectively on video data. Only 12 of the 50 papers validated their algorithms on actual participants, whereas the remaining 38 studies validated their approaches on databases alone. Whilst datasets are a valid and more accessible method of testing DL HPE models, sport is dynamic and unconstrained, and therefore demands realism as factors like occlusions, different camera resolutions, weather conditions, clothing (i.e., sports kit and equipment) and unique poses due to individual differences can impact tracking [9, 76]. These trends continued to prevail within the studies found in the supplementary search as only one study validated their approach on live participants; however, this was not in a laboratory-based setting and or an in-game scenario which affects the generalisation of the findings [73].

### Movement Skill Analysis

Applying DL HPE to perform movement skill analysis on athletes non-invasively (i.e., without the need for additional markers or sensors) makes these methods more ecologically valid (i.e., a more natural form of measuring) compared to traditional quantitative methods [77]. OpenPose emerged as the most popular algorithm for movement skill analysis with 6 studies including 2 comparative studies using this model either on its own or with an additional model (see Table 5). Studies compared OpenPose with other DL HPE algorithms in paddleball and surfing [25, 39], whilst two others used OpenPose alongside an additional model to improve the accuracy of their approaches in skiing and basketball [19, 21]. All these approaches achieved sufficient accuracy to be applied within an athlete development program in their respective sports and can provide coaches with non-invasive movement analysis data to inform their decision making on their athlete’s movement skill training [19, 21, 25]. These results demonstrate that OpenPose alone or combined with another model has the potential to positively affect the movement skill acquisition process in a variety of sports. However, three studies were trained on small private datasets containing limited variations of athletes and backgrounds, which can affect performance when applied in environments with complex backgrounds [18, 36, 76]. The overwhelming tendency to validate these approaches in small private datasets and not in real participants in a performance context is a key limitation through the ED lens. As argued in previous research analysis performed by machine learning and computer vision systems must occur in representative performance environments that preserve natural interactions between athlete, task and environment [74]. Thus, the ecological validity of these movement analysis tools tested on small private and ambiguous datasets remains questionable. To properly evaluate if systems trained on ambiguous datasets are suitable for athlete development, developers should release their training data. While these systems may offer valuable insights, their practical application cannot be fully considered without access to the source data.

Contrastingly, 2 studies used OpenPose in real-life participants; one of these studies used OpenPose alone to conduct 2D swing analysis in baseball in 10 male university athletes [27], whilst the other used OpenPose with an additional model and trained on a public dataset to assess the 3D centre of mass behaviour of 12 elite sprinters [31]. Overall, applying OpenPose, with or without additional models, to real-life participants yielded significant accuracy. These findings echo the insights of a previous systematic review indicating a trend for using general-purpose algorithms in sports due to limited sport-specific alternatives, particularly in less represented sports like paddleball, Alpine skiing, and surfing [2]. A similar trend was found in the only study found in the supplementary search which validated a publicly available model (RTMPOSE) in real participants to quantify movements in a 3 × 3 basketball game [73]. However, the approach outlined used a private dataset and large multi-camera system, which while more accurate and reliable compared to single camera approaches, its large expense and private dataset make it inaccessible for practitioners [73].

There was also a diverse range of bespoke DL HPE algorithms used to achieve movement skill analysis present in this systematic review. Bespoke algorithms offer tailored solutions to difficult analysis problems but require creation 'from the ground up' by developers. From an application perspective, bespoke algorithms may not be as appropriate for athlete development programmes with no access to developers as they are expensive and time-consuming to create. GolfPose and G-20 Pose are bespoke HPE algorithms that perform full-swing analysis in golf offering golf practitioners the ability to perform movement skill analysis non-invasively on the golf course which is advantageous compared to traditional approaches [26, 36]. GolfPose offers practitioners more insights into performance with its ability to track the club and provide club metrics (i.e., club head speed and shaft angle) allowing practitioners to make more informed decisions. The most popular use of bespoke DL HPE algorithms for movement skill analysis was in water sports (i.e., swimming and diving); these studies used a combination of public and private datasets to train and validate their 2D DL HPE algorithms [30, 37, 38]. Furthermore, SwimmerNET is a 2D DL HPE algorithm which was also trained on a private dataset but was validated on elite swimmers and was the only bespoke approach to be validated in this manner [22]. The authors' approach to validation is highly representative so practitioners can be confident when applying this approach in practice [22].

While the previous systematic review correctly identified the prevalence of private datasets as a barrier to reproducibility, this review advances these findings by providing a critical and pragmatic analysis of why current data approaches are insufficient for sports science practitioners [2]. Currently researchers and developers either rely on small, private datasets that are often not representative of real-world sporting scenarios, or they utilise large, publicly available datasets like COCO and UCF Sport. Whilst private dataset creation may sometimes be necessary for underrepresented sports like paddleball [25], we argue these current large datasets lack the contextual factors necessary for robust sport-specific applications as most of their content does not capture the unique human factors present in real sporting contexts. This review proposes an action plan calling for a collaborative effort between sports and computer science communities to create open-access, large-scale benchmark datasets for sport such as football, basketball, and tennis. However, these datasets should be available for researchers and applied practitioners as making them a commercial entity adds yet more barriers to the future implementation of DL HPE in sport. These datasets must be contextually rich, including a wide range of skill levels, diverse performance environments, and high-density, multi-person scenarios characteristic of team sports. Some pilot studies have been conducted on the use of generative AI for synthetic visual datasets (i.e., like those used to train DL HPE models) [78]. While this approach remains untested in training DL HPE algorithms applied in sport it could offer a long-term solution to the lack of representative training data currently available for DL HPE algorithms. Although generative AI could be used to create training data it cannot be used as a HPE technique itself as that is not its purpose.

While this review primarily focuses on movement skill analysis at an individual level, there is significant potential for DL HPE to extend into tactical analysis. Tactical analysis would require robust multi-person HPE systems capable of accurately tracking multiple athletes and analysing their spatial and temporal interactions. However, current limitations, including computational and hardware requirements and occlusions in team sports, present challenges for practical applications especially for 3D multi-person pose estimation [79]. These same challenges still occur in more recent studies identified within the supplementary search [71]. Overall, the discussed DL HPE approaches to movement skill analysis in sport all offer sufficient accuracy to be applied within athlete development programs with little adaptation required. Therefore, they could all have positive effects on athlete performance, but the effects of their long-term implementation on coaching and athlete performances remain unexplored.

### Action Recognition

Bespoke algorithms were the most popular approach to creating DL HPE action recognition systems, with two studies creating action recognition systems that can be applied across multiple sports (i.e., golf, diving, weightlifting, horse riding, and running) [1, 45]. These algorithms were trained and validated on very large and diverse public datasets containing a variety of general and sport specific scenarios whilst also utilising publicly available algorithms [1, 45]. These approaches to action recognition provide coaches with reproducible and accessible notational analysis about actions performed, allowing a variety of practitioners across multiple sports to make informed coaching decisions based on accurate performance data. The application of DL HPE for action recognition in racquet sports was also a popular application setting. One study compared multiple 2D DL HPE algorithms to create an automatic performance analysis tool in table tennis [43]. Another study developed a 3D DL HPE to create an action recognition system in tennis [44]. These systems offer racquet sport practitioners accurate automatic performance analysis systems in 2D or 3D which can provide in-depth analysis on athlete performance and save organisations time and money by automating this process. These algorithms are formed from publicly available models (i.e., OpenPose, inverse transformer models and TCNs) and trained on large public datasets containing general and sport specific images, making these algorithms valid and reproducible approaches for applied practitioners [44, 45].

The most popular setting for DL HPE for action recognition was in team sports (see Table 6); two studies created bespoke automatic performance analysis tools in basketball, with both studies choosing to train and validate their approaches on small but highly sport specific private datasets [48, 49]. These approaches save time for practitioners by automating analysis, providing accurate performance data to inform decisions, but are limited to single-person HPE which reduces the contextual understanding in team sports like basketball. Moreover, two studies created action recognition systems in ice hockey [41, 46], whilst another created a similar algorithm in volleyball [47]. These systems cannot quantify all actions, so currently could not fully replace manual notational analysis, but provide first steps of using DL HPE to create action recognition systems in their sports. Again, these algorithms are comprised of publicly available models, and while providing practitioners with accessible and reproducible automated data collection tools, these approaches share the same limitation as previous applications of DL HPE in team sports as they are single person focused, potentially losing the broader context of the match [41, 46, 48, 49]. From an ED perspective this is a key limitation of current team-sport applications of action recognition DL HPE, i.e. they lack key environment constraints present in all team sports, namely the dynamic interactions with other players which shape the emergence of unique movement patterns.

The supplementary search identified an additional five studies that are classified as action recognition systems [67–71]. These studies again continued to follow the same trends as previous literature found in the original search. All studies used 3D HPE and used datasets to validate their approach; however training and validation was typically conducted on large and representative publicly available datasets. Although all approaches were bespoke algorithms, many were created from several layers of publicly available models, meaning whilst these approach may be difficult for practitioners to adopt in their current form and all need adaptations before being implemented into coaching pedagogy, most aspects are all publicly accessible.

The popularity of custom DL HPE algorithms for action recognition stems from the need to first detect and track athletes and then categorize actions. From an application perspective, practitioners are interested in what can give them the most accurate and viable results, so whether an algorithm is general-purpose or bespoke does not matter. Building upon action recognition, action localization offers the potential to identify the precise spatial and temporal onset of actions, which could provide more granular insights for coaches and analysts. While some of the reviewed studies could be adapted and validated to perform action localisation, its practical implementation is limited by the need for higher temporal resolution and more comprehensive datasets. Therefore, we call for developers to work in tandem with sports practitioners to again focus on creating larger, more diverse datasets to support and increase a wider variety of DL HPE applications in sport. This systematic review outlines to applied practitioners that there are publicly available algorithms that could be paired with publicly available datasets to achieve action recognition in a wide array of sport contexts, but the further development of multi-person DL HPE in sport is crucial to give practitioners performance data that are contextualized to the whole match.

### Augmented Coaching Tools

The most popular approach to augmented coaching tools using DL HPE was with bespoke algorithms because of the multiple models required to create an augmented coaching tool (i.e., tracking and detection model, classification model, and feedback model). The most popular application was to create augmented coaching tools that can aid in the learning of different martial arts in 2D and 3D [50, 54, 59, 60]. These approaches all presented sufficient accuracy to be applied in a home or training environment to provide extrinsic feedback that allows athletes to adjust their martial art movement skills without the need for a coach. An individual could apply one of these systems in their home environment, with the potential to demonstrate movement skills, provide performance feedback, and theoretically lead to performance improvements without the need for a coach [59]. While these studies focus on improving movement skill through augmented feedback, the backbone of these systems could potentially extend to broader applications, such as rehabilitation monitoring and assisting in the officiating of sport, though these applications remain underexplored.

Due to the underrepresentation of these sports in public datasets, most studies used small, non-representative private datasets to train and validate their approaches, which, as mentioned previously, reduces the reproducibility and potential applicability of these methods [54, 59, 60]. This tendency continued in the only augmented coaching tool found in the supplementary search created a private climbing dataset to train and validate their route helper in rock climbing. Again, a prevailing trend within this systematic review is the absence of longitudinal studies assessing the long-term implementation of DL HPE in sport. The absence of long-term studies means the performance effects of DL HPE in sport remain unknown, which may deter practitioners from adopting this technology [2]. The applicability of pose estimation depends on factors such as movement complexity, environmental constraints, and dataset quality. Future research should prioritize demonstrating the performance differences resulting from the long-term implementation of DL HPE in athlete development programs.

Augmented coaching tools were the most popular application type in this systematic review to validate their approaches on actual sport participants, with racquet sports being the most common (see Table 7). Augmented coaching tools were created in badminton and table tennis [53, 61], with them being validated on 15 badminton players and 10 table tennis players respectively. Both approaches were trained on extensive, varied, and publicly accessible datasets, suggesting that they may yield superior performance when integrated into athlete development programs. This review highlights that the effectiveness of augmented coaching tools often hinges on the quality and representativeness of the datasets used for training and validation, as well as the sophistication of the methods employed. Bespoke models tailored to specific sports can yield high accuracy, but their reliance on private datasets limits reproducibility.

The application of DL HPE in table tennis demonstrated that it could increase the enjoyment of amateur athletes by providing more information during performance and paradoxically teaching advanced players to adapt their pose to conceal the resulting ball path, all without the need for coaching input [61]. Furthermore, both methodologies can act as an automatic notational analysis tool, reducing practitioner workload and enriching coaches’ decision-making with data, whilst supporting athlete development of core badminton skills [53, 61]. Overall, the utilization of DL HPE for the creation of augmented coaching tools was found to be the second most popular form of application in this systematic review, with the most popular application being in martial arts contexts offering athletes extrinsic feedback on movement skill without coach intervention.

This review of augmented coaching tools provides a significant contribution to knowledge by establishing this as a distinct and coherent application strategy chosen by developers. While prior reviews have noted individual examples of coaching aids in sports like badminton or yoga, they failed to group them as a unique class of application, meaning trends, challenges, and the practical readiness of these tools were largely unexamined [2]. Our analysis reveals that these tools are most prevalent in individual skill-based sports like martial arts and table tennis, but their development is critically hampered by a reliance on small, private datasets that lack the key contextual factors that are crucial for robust algorithm development. By systematically identifying the universal absence of longitudinal validation studies across the applications of DL HPE, our review establishes that the performance efficacy of these coaching tools remains theoretically promising but empirically unproven.

From a theoretical perspective, previous research advocates that the future implementation of HPE in sport should be conducted though the collaboration of developers, athletes and most importantly coaches but many of these augmented coaching tools remove the need for a coach, which which could have long term impacts on performance [74]. The design of many of these coaching tools can be appraised against an ecological framework to outline implications of its application in athlete development programs. All of these augmented coaching tools in some way provide prescriptive extrinsic feedback risks that create a dependency on the technology, potentially hindering an athlete's ability to self-organise and attune to their own intrinsic feedback during performance [74]. Future research should therefore aim to develop coaching tools not as rigid error-correctors, but as systems that manipulate task constraints or provide outcome-based information with their design grounded in an ecological framework.

### Systems to Support the Officiating of Sport

Recent advances in HPE mean it is now possible to enhance the officiating of sporting events even in challenging scenes when occlusions are common and the speed is high, thus enhancing the visual perception of officials through DL HPE [62]. Beyond officiating, these advancements could also support applications such as real-time tactical analysis, injury prevention through monitoring collision dynamics, and automated player tracking in team sports, which remain underexplored but hold significant promise. Rugby was the most popular sport for the application of DL HPE to support officials, with three studies utilising 3D DL HPE in rugby to make the sport safer for players by reducing the risk of acute injuries (e.g., broken bones) and long-term injuries (e.g., neurological conditions) [63, 65, 66]. Despite the promise of these advancements, significant validation challenges remain. Only one of the reviewed studies validated its approach in real-life participants [63], and even then, this was in a non-contextual sporting environment (i.e., a biomechanics laboratory). This setting lacks key environmental factors such as other players, crowds, and weather, reducing transferability. Furthermore, this validation approach which included 2 participants is not representative of performance conditions as in a real rugby scenario the model would need to track up to 30 players simultaneously from multiple camera angles. This leap in scale introduces the key challenges of multi-person estimation: it drastically increases the required computing power and, more critically, exposes the model to severe and chaotic player-on-player occlusion, which is constant in a match but absent in a simple 2-person trial [80]. Moreover, the need to process this high volume of data in real-time makes the system vulnerable to dropped frames, which can cascade into catastrophic tracking failures. These computational and algorithmic complexities must be addressed, and future studies should therefore prioritize validating algorithms in real-world match scenarios to ensure models can perform effectively in such dynamic and unpredictable settings. It must be stated these issues need to be solved by developers and researchers before these approaches are accessible to applied practitioners.

The reliance on private datasets for multi-person HPE in recent rugby studies [63, 65, 66] significantly limits reproducibility. However, this approach was likely necessitated by the inadequacy of existing public datasets to train complex multi-person HPE models in real-world contact sports. For instance, while the large-scale Human3.6 m dataset provides multi-person 3D skeletons, its scenarios (e.g., dancing, social games) lack the high-velocity, high-impact, and chaotic occlusions specific to rugby. Similarly, the MADS dataset [81] includes relevant high-velocity sporting actions, but its data are limited to one or two players, failing to capture the large-scale game scenarios of rugby, which can involve up to 30 players simultaneously. This gap between public data and practical requirements pushes practitioners to create bespoke datasets, which not only limits reproducibility but may also contribute to the high error levels reported in these collision-heavy contexts. This highlights a critical need for future open-access datasets that feature large player counts, frequent collisions, and varied environmental conditions to improve the accuracy and generalisability of DL HPE systems.

## Conclusion

This systematic review aimed to assess the applications of DL HPE in sport by answering 3 research questions, (1) what are the current and potential applications of this technology?, (2) what published approaches are accessible to practitioners regarding public availability and reproducibility?, and (3), what are the human factors relating to its application in sport? Specifically, this systematic review categorised DL HPE into 4 distinct applications: movement skill analysis, action recognition, augmented coaching tools, and officiating tools. In this systematic review, bespoke multi-model algorithms made up of public models were the most popular approach to DL HPE which is of benefit to their accessibility, but most studies chose to validate and train algorithms on private datasets, which reduces the reproducibility of these algorithms and hinders the future applications of DL HPE in sport. However, the biggest drawback to the current applications of DL HPE in sport is that, to our knowledge, no study has empirically analysed the effects of longitudinal implementation of DL HPE in athlete development programs for any task (i.e., movement skill analysis, action recognition, augmented coaching tool, or officiating aids). From a practitioner’s perspective, this may limit their willingness to adopt these tools and it is unclear if it would lead to performance adaptations in their athletes. Therefore, to advance the field of sports science and fully harness the potential of DL HPE, we urgently call upon researchers, developers, and practitioners to prioritise the creation of open, standardized datasets, implement reproducible methodologies, and conduct longitudinal studies that empirically validate these technologies in real-world athlete development programs.

## Data Availability

The data used to form this systematic review can all be accessed publicly using the search terms outlined within the manuscript. In addition, data can be shared upon reasonable request by contacting the corresponding author.

## Abbreviations

3DCNN: 3D convolutional neural network
Acc: Accuracy
ANN: Artificial neural network
AP: Average precision
AR: Average recall
ARHN: Action recognition Hourglass Network
AUROC: Area under the receiver operating characteristic curve
BFAP: Body feature alignment based on pose
CNN: Convolutional neural network
CPM: Convolutional pose machine
CV: Coefficient of variation
DCNN: Deep convolutional neural network
DNN: Deep neural network
DR: Detection rate
FCN: Fully convolutional network
FNN: Fuzzy neural network
GCNN: Graph convolutional neural network
HRNet: High-resolution network
LSTM: Long short-term memory
LT: Learnable triangulation
MAE: Mean absolute error
MAP: Mean average precision (also seen as mAP)
MCC: Matthews correlation coefficient
MPE: Mean percentage error
MPJPE: Mean per joint position error
MSE: Mean squared error
PAFS: Part affinity fields
PCA: Percentage of correct angles
PCK: Percentage of correct key points
PCKP: Percentage of correct keypoints-proximal
PCP: Percentage of correct point
PHRNN: Part-based hierarchical recurrent neural network
Prec: Precision
PRNN: Piecewise recurrent neural network
R-CNN: Region-based convolutional neural network (also seen as RCNN, R-Cnn)
Rec: Recall
RMPE: Regional multi-person pose estimation
RMSE: Root mean squared error
RNN: Recurrent neural network
RPN: Region proposal network
SD: Standard deviation
SEE: Standard error of estimate
SMAPE: Symmetric mean absolute error
SPPE: Single-person pose estimation
ST-GCN: Spatio-temporal graph convolutional network
STN: Spatial transformer network
STRM: Spatial–temporal relation module
T-CNN: Temporal convolutional neural network
TCN: Temporal convolutional network
TSDNN: Time series deep neural network
TSN: Temporal segment network
